# Comparison and Temporal Trends of Three Groups with Cryptococcosis: HIV-Infected, Solid Organ Transplant, and HIV-Negative/Non-Transplant

**DOI:** 10.1371/journal.pone.0043582

**Published:** 2012-08-24

**Authors:** Emily W. Bratton, Nada El Husseini, Cody A. Chastain, Michael S. Lee, Charles Poole, Til Stürmer, Jonathan J. Juliano, David J. Weber, John R. Perfect

**Affiliations:** 1 Department of Epidemiology, University of North Carolina at Chapel Hill, Chapel Hill, North Carolina, United States of America; 2 Department of Medicine, Duke University, Durham, North Carolina, United States of America; 3 Department of Medicine, Vanderbilt University Medical Center, Nashville, Tennessee, United States of America; 4 Department of Medicine, Brigham and Women’s Hospital, Boston, Massachusetts, United States of America; 5 Department of Medicine, University of North Carolina at Chapel Hill, Chapel Hill, North Carolina, United States of America; Baylor College of Medicine, United States of America

## Abstract

**Background:**

The Infectious Disease Society of America (IDSA) 2010 Clinical Practice Guidelines for the management of cryptococcosis outlined three key populations at risk of disease: (1) HIV-infected, (2) transplant recipient, and (3) HIV-negative/non-transplant. However, direct comparisons of management, severity and outcomes of these groups have not been conducted.

**Methodology/Principal Findings:**

Annual changes in frequency of cryptococcosis diagnoses, cryptococcosis-attributable mortality and mortality were captured. Differences examined between severe and non-severe disease within the context of the three groups included: demographics, symptoms, microbiology, clinical management and treatment. An average of nearly 15 patients per year presented at Duke University Medical Center (DUMC) with cryptococcosis. Out of 207 study patients, 86 (42%) were HIV-positive, 42 (20%) were transplant recipients, and 79 (38%) were HIV-negative/non-transplant. HIV-infected individuals had profound CD4 lymphocytopenia and a majority had elevated intracranial pressure. Transplant recipients commonly (38%) had renal dysfunction. Nearly one-quarter (24%) had their immunosuppressive regimens stopped or changed. The HIV-negative/non-transplant population reported longer duration of symptoms than HIV-positive or transplant recipients and 28% (22/79) had liver insufficiency or underlying hematological malignancies. HIV-positive and HIV-negative/non-transplant patients accounted for 89% of severe disease cryptococcosis-attributable deaths and 86% of all-cause mortality.

**Conclusions/Significance:**

In this single-center study, the frequency of cryptococcosis did not change in the last two decades, although the underlying case mix shifted (fewer HIV-positive cases, stable transplant cases, more cases with neither). Cryptococcosis had a relatively uniform and informed treatment strategy, but disease-attributable mortality was still common.

## Introduction

*Cryptococcus neoformans* is an invasive mycoses that can cause meningoencephalitis, particularly among those who are immunocompromised, but in some cases it can infect immunocompetent individuals [1]. The 2010 IDSA Cryptococcal Guidelines defined three distinct risk groups for induction treatment of cryptococcosis [2]: (1) HIV-positive; (2) transplant recipients; and (3) a heterogeneous group with neither of these conditions (i.e., HIV-negative/non-transplant). A major component of this review was to describe outcomes of recent management of these three groups. During this study, four important factors were in play that justified our decision to use the broad 14-year study period in order to maximize cohort size. First, HAART became readily prescribed in 1996 with supportive evidence of the superiority of combination antiretroviral therapy over monotherapy in reducing AIDS morbidity and mortality [3]. Second, lipid products of amphotericin B, for patients with renal impairment or unacceptable toxicity that prevent the use of conventional amphotericin B, were in use since their initial FDA approval in November, 1995 [4]. Third, in 2000 the original IDSA Guidelines were published as a standard of treatment [5]. Fourth, there was an active Infectious Disease group at our institution with a particular interest in the pathogenesis and treatment of cryptococcosis.

The three risk groups defined by the IDSA and corresponding depths of information supporting treatment guidelines have been shaped through their individual patterns of emergence over time. In the last two decades, HIV-positive populations with cryptococcosis have been the most widely studied group [6], [7], [8], [9], [10], [11], [12], [13], [14], [15], [16], [17], [18] and have received greater attention recently due to the recognition that cryptococcosis incidence in this group remains high, particularly in the AIDS-infected population in sub-Saharan Africa [19]. Starting in the 1960–1980’s, use of immunosuppressive medications to treat severe diseases or for solid organ transplantation has increased the pool of patients susceptible to *Cryptococcus*. In the late 1990’s, *Cryptococcus gattii* emerged in Vancouver Island, British Columbia, resulting in an outbreak of infections in both immunosuppressed and immunocompetent hosts [20], [21]. The HIV-negative cryptococcosis patient group had been excluded from clinical review for several decades but has gained more attention recently [22], [23], [24], [25], [26], [27]. Cryptococcal patients who are HIV-negative, particularly those who have few or no underlying risk factors (i.e., “apparently immunocompetent”), may experience more of a delay in time to presentation and diagnosis than HIV-positive or transplant recipient patients [26]. In particular, recent evidence has shown that HIV-negative, non-immunosuppressed cryptococcal meningitis patients suffered higher mortality rates than HIV-positive patients [25].

Due to its rare occurrence, prospective observational studies of this disease are logistically problematic. Interventional approaches have historically been based on expert opinion and retrospective cohort studies more than a decade old, with few representing HIV-negative populations and comparatively developed countries [26], [28], [29], [30]. In this relatively large, retrospective single-center study, our goal was to provide an in-depth look at how cryptococcosis was managed clinically in the HIV-positive, transplant recipient and HIV-negative/non-transplant patient groups in order to improve our understanding of this disease.

## Methods

### Objectives

The goals of this study were to describe trends in cryptococcosis symptoms, diagnosis, treatment and mortality through a 14-year study period (1996–2009) within the context of the three groups defined by the IDSA Guidelines.

### Participants

We identified all consecutive adult patients (≥18 years old) discharged from DUMC with International Classification of Diseases, 9th Revision (ICD-9) diagnosis codes of cryptococcosis (117.5), and cryptococcal meningitis (321.0) between January 1, 1996 and October 31, 2009 through electronic medical records. Eligible subjects had confirmed cryptococcal disease and a sufficient medical record (electronic and/or paper chart) available for review. A cryptococcosis case was confirmed by having ≥1 of the following: positive cerebral-spinal fluid (CSF) cryptococcal antigen (CRAG) or fungal culture, direct histological examination of tissue or fluid with characteristic yeast forms of *Cryptococcus*, positive serum cryptococcal antigen test with a consistent disease process or positive culture from blood or pulmonary sites.

### Description of Investigations

Demographics, presenting symptoms (including duration), and underlying conditions at the time of diagnosis were collected. Clinical differences examined included: presentation and duration of symptoms, microbiological evidence of cryptococcal disease, and initial antifungal treatment. Clinical isolates were not typed in this study. *C. gattii* is rare in the Southeastern U.S. with only one identified clinical case in an immunocompromised adult [31]. Serotype A (*C. neoformans* var. *grubii*) predominates the region of our study [32]. Follow-up visit information relevant to cryptococcosis (laboratory testing, clinic visits, and readmission) was also captured. Patients were followed from the date of diagnosis and/or admission until loss-to-follow-up, death, or until the end of the study period. In order to assess one-year mortality prevalence for all patients, we obtained data on survival and mortality up to one year after their date of cryptococcosis diagnosis from the Duke Data Support Repository (DSR), which uses the Social Security Administration death index, the Tumor Registry and The Duke Information System for Cardiovascular Care death data to report mortality status. Investigators recorded all information on a standardized abstraction form developed in collaboration with epidemiologists and clinicians.

We report on annual changes in frequency of cryptococcosis diagnoses, treatment, and outcomes including: overall mortality through one year, deaths attributable to cryptococcosis, and occurrence of immune reconstitution inflammatory syndrome (IRIS). Defined and determined by a panel of experts at DUMC, death was attributable if patients experienced conditions directly related to cryptococcal disease, such as: increased central nervous system (CNS) pressure, persistence or relapse of infection, while receiving initial induction treatment or due to organ failure during treatment for cryptococcosis. The criteria used to identify IRIS, adapted from Singh and Perfect (2007), included clinical or radiographic manifestations consistent with an inflammatory process, such as contrast enhancing lesions on imaging studies (CT/MRI), combined with symptoms that occurred during receipt of appropriate therapy and could not be explained by newly acquired infection, and at least one of the following: (1) negative results for cultures or stable/reduced biomarkers for the initial fungal pathogen during diagnostic work-up for the inflammatory process, (2) CSF pleocytosis >5 WBC/mm3, (3) Increased ICP, (4) histopathology showing granulomatous lesions, or (5) unexplained hypercalcemia [33].

Central nervous system (CNS), pulmonary and ‘other’ cryptococcosis patients were collapsed into two categories based on specific indicators described in the 2010 IDSA Treatment Guidelines [2]: severe disease (evidence of CNS involvement, or cryptococcemia or dissemination with evidence of high fungal burden based on serum CRAG ≥1∶512) or non-severe disease.

### Ethics

This study was approved by both the Duke University Medical Institutional Review Board (IRB) and the University of North Carolina at Chapel Hill Biomedical IRB. Both named IRBs waived the need for informed consent for this study. This research met criteria for a waiver of informed consent according to Title 45, Code of Federal Regulations (CFR) Part 46.116(d).

### Statistical Methods

All data was entered into Microsoft Office Access (2007) and analyses were performed using SAS v9.2 (SAS Institute, Cary, NC). Variables were examined using descriptive statistics and stratified based on the three groups and/or severity of cryptococcosis as needed. Where appropriate, the Student’s t-test was used to test the difference of two means and the Kruskal-Wallis test was used for the difference between medians for non-parametric continuous data. Chi-square (Χ^2^) tests were used to examine differences between categorical frequency distributions. The statistical significance level of alpha (α) equal to 0.05 was used for each two-tailed test performed, thus a “significant” result refers to a p-value <0.05.

## Results

There were 223 study patients identified; 16 were excluded due to the following reasons: unable to locate chart (n = 1), transferred out of care prior to diagnostic test results and did not receive treatment at DUMC (n = 4), patient <18 years (n = 1), and the patient did not have verified cryptococcal disease (n = 10). A total of 207 patients were used for analysis. The majority of cases were CNS disease (61%), followed by pulmonary (34%) and other sites (Table 1). Consistent across the three clinical groups, nearly two-thirds of the cohort were male (65%) and African Americans were more prevalent among HIV-positive patients than the other two groups (Table 1). HIV-positive patients were significantly younger than the other two groups.

**Table 1:** Patient characteristics at baseline.

				Underlying Condition^a^						
Category	Subcategory	Total		HIV		Transplant		HIV−/Trans-b		
		N	(%)	n	(%)	n	(%)	n	(%)	p-value^c^
Primary Diagnosis										
	CNS	126	(61)	74	(86)	18	(43)	34	(43)	>0.05
	Pulmonary	71	(34)	9	(10)	24	(57)	38	(48)	
	Other	10	(5)	3	(4)	0	(–)	7	(9)	
Disease severity										
	Severe	131	(63)	74	(86)	18	(43)	39	(49)	>0.05
	Non-severe	76	(37)	12	(14)	24	(57)	40	(51)	
Demographics										
	Male Gender	135	(65)	55	(64)	28	(67)	52	(66)	>0.05
	Black Race	106	(53)	69	(80)	13	(31)	24	(30)	>0.05
	Age (yrs)^d^	47	(15)	40	(9)	50	(14)	54	(18)	**<0.05**

Primary diagnosis, disease severity, basic patient characteristics and underlying condition of cryptococcosis patients at DUMC (N = 207).

a There were 86 patients in the HIV group, 42 in transplant and 79 in HIV-negative, non-transplant.

b HIV-negative and non-transplant.

c Cochran Mantel-Haenszel Chi-square test for a general association between the three groups; Kruskal-Wallis test for difference between median age was used.

d Instead of n (%), mean (STD) are shown for age.

**Figure 1:**
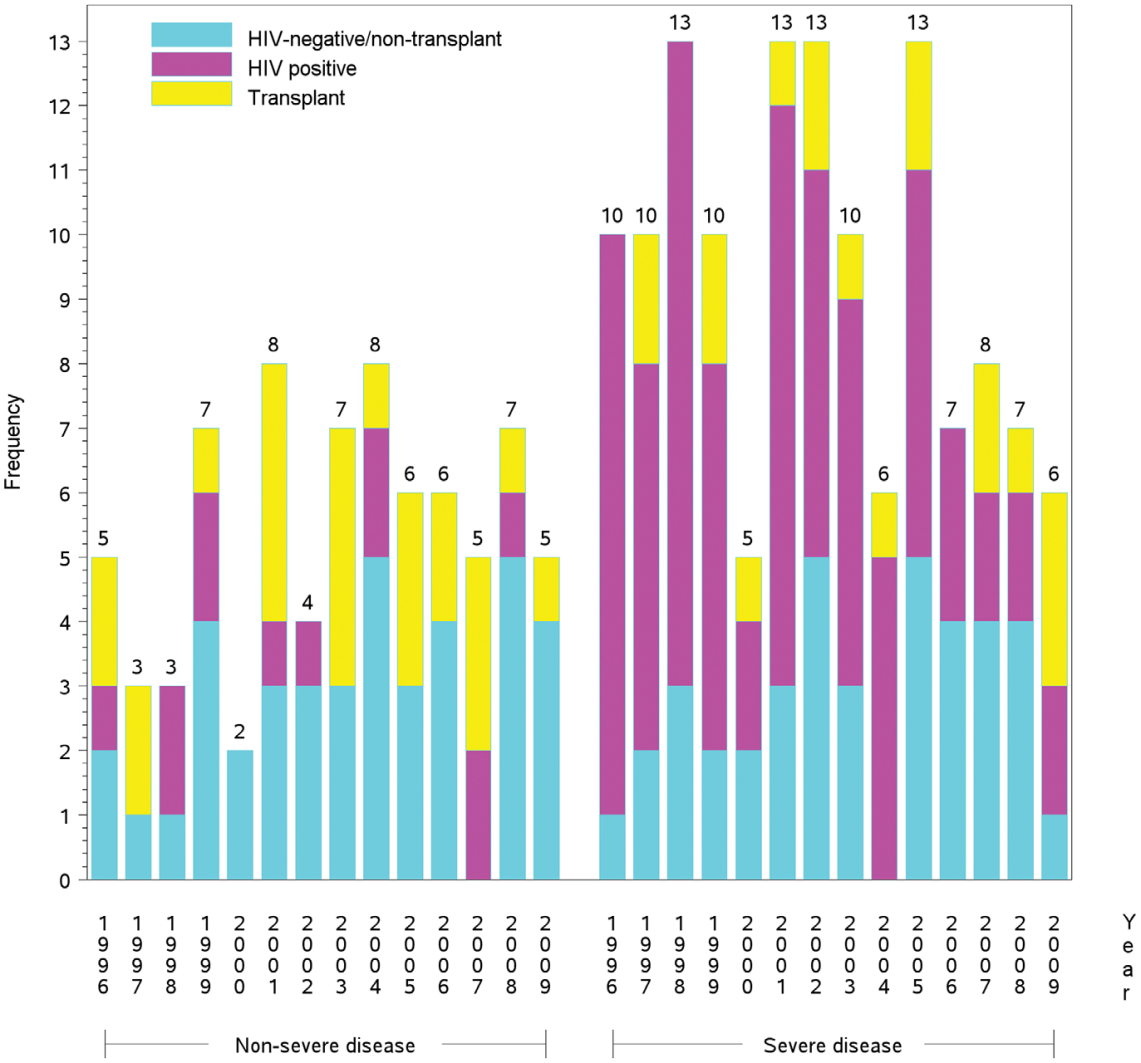
Annual cases. Annual frequency of severe and non-severe cryptococcosis cases according to underlying condition (N = 207).

Overall annual case frequencies of cryptococcosis did not significantly change over time (Figure 1). During the study period, the number of transplant patients per year averaged three (range, 0–5 cases/yr.).The frequency of HIV-infected cases averaged six annually (range, 2–12 cases/yr.). Among HIV-negative, non-transplant cases there was a slight increasing trend with time; the annual average number of cases was six (range, 3–9 cases/yr.). Although the total cases have remained relatively steady (∼15/yr.), there appeared to be a shift to a decreasing proportion of HIV-positive patients with a concomitant increase in HIV-negative cases. HIV-positive patients accounted for half of all cases in the first seven years of this study then fell to less than one-third in the latter seven years.

### Within-group Observations

Within the HIV-positive patient group (n = 86), 74 (86%) had CNS disease, 9 (11%) had pulmonary cryptococcosis and three patients had another form of cryptococcosis (Table 1). Twenty-seven (31%) patients were newly identified as positive for HIV infection during their hospital admission for cryptococcosis. CD4 counts were available for 62 (72%) patients during hospitalization (median, 22 cells/µL; range: 1–300 cells/µL). Forty-two percent of HIV-positive patients with severe (n = 31) and non-severe (n = 5) cryptococcosis reported current or previous exposure to HAART therapy, but management compliance was heterogeneous. Prevalence of reported HAART exposure did not differ significantly comparing earlier (1996–2002) and later cases (2003–2009). During admission 18 patients (21%) continued their known HAART therapy, four patients (5%) changed to another regimen, six patients (7%) continued their current therapy but changed their regimen at the time of discharge and six patients had their therapy held on admission. Seventeen patients (20%) had no confirmed previous exposure to HAART; eight died with cryptococcal disease-related deaths. Thirty-five patients (41%) were started newly on HAART therapy at the time of or after discharge. The median time-to-start of HAART therapy was 67 days after the start of induction therapy.

Among transplant recipients (n = 42), 18 (43%) had CNS disease (severe) and 24 (57%) had pulmonary disease (non-severe; Table 1). The majority of transplants were renal (n = 17; 4 included pancreas) followed by cardiac (n = 11) and pulmonary (n = 9). The median time from transplant to diagnosis of cryptococcosis (n = 41) was 26 months (Inter-Quartile Range [IQR]: 10–56 months). Over one-third (n = 16) of transplant recipients had renal insufficiency at the time of diagnosis (Table 2), but only two patients (11%) with severe disease experienced a >50% decrease in Glomerular Filtration Rate (GFR) during induction treatment. Current steroid exposure at the time of cryptococcosis was high (93%), and 19 patients (24%) had their immunosuppressive therapy stopped or changed at the time of their cryptococcosis diagnosis. Of the 39 patients taking steroids prior to cryptococcosis diagnosis, 37 (95%) had dose information. The median daily dose was 10 mg of prednisone or prednisone equivalent (range: 4–30 mg/day). Most of these patients had been on extended immunosuppressive therapy. The median duration of immunosuppression (n = 39) was 19 months (IQR: 8–56 months).

**Table 2:** Presenting symptoms and risk factors of cryptococcosis disease (N = 207).

		Severe disease (n = 131)^a^								Non-severe disease (n = 76)^a^						
Presentation	Characteristic	HIV		Transplant		HIV−/Trans-			*p<0.05	HIV		Transplant		HIV−/Trans-		*p<0.05
		n	(%)	n	(%)	n		(%)		n	(%)	n	(%)	n	(%)	
Symptoms																
	No symptoms	1	(1)	0	(–)	0		(–)		1	(8)	10	(42)	9	(23)	
	Altered mental status^b^	22	(30)	7	(39)	17		(44)		0	(–)	1	(4)	0	(–)	
	Headache	54	(73)	9	(50)	17		(44)	*	2	(17)	3	(13)	2	(5)	
	Cough	15	(20)	1	(6)	3		(8)		7	(58)	3	(13)	11	(28)	*
	Shortness of breath	10	(14)	1	(6)	5		(13)		6	(50)	5	(21)	14	(35)	
	Night sweats	5	(7)	2	(11)	1		(3)		4	(33)	2	(8)	4	(10)	
	Fever	40	(54)	7	(39)	12		(31)		6	(50)	4	(17)	15	(38)	
	Nausea	33	(45)	11	(61)	6		(15)	*	2	(17)	5	(21)	5	(13)	
	Vomiting	28	(38)	8	(44)	5		(13)	*	3	(25)	3	(13)	3	(8)	
	Seizures	8	(11)	0	(–)	3		(8)		0	(–)	0	(–)	1	(3)	
Condition/risk factor																
	Renal insufficiency	3	(4)	10	(56)	5	(13)		*	3	(25)	6	(25)	6	(15)	
	Liver insufficiency	1	(1)	0	(–)	6	(15)		*	1	(8)	0	(–)	2	(5)	
	Hematologic malignancy^b^	0	(–)	1	(6)	7	(18)		*	0	(–)	0	(–)	7	(18)	*
	Non-hematologic Malignancy	0	(–)	0	(–)	2	(5)			1	(8)	0	(–)	3	(8)	
Immunosuppressants																
	Corticosteroid	4	(5)	17	(94)	19		(49)	*	1	(8)	22	(92)	12	(30)	*
	Calcineurin inhibitor	0	(–)	15	(83)	0		(–)	*	0	(–)	21	(88)	2	(5)	*
	Mycophenolate mofetil	0	(–)	13	(72)	2		(5)	*	0	(–)	10	(25)	2	(5)	*
	Azathioprine	0	(–)	2	(11)	1		(3)	*	0	(–)	8	(33)	1	(3)	*
	Methotrexate	0	(–)	1	(6)	1		(3)		0	(–)	3	(13)	3	(8)	
	Monoclonal antibodies	1	(1)	2	(11)	3		(8)		0	(–)	0	(–)	2	(5)	
	Sirolimus	0	(–)	2	(11)	0		(–)	*	0	(–)	2	(8)	0	(–)	

Patients are stratified by disease severity at initial presentation and underlying condition.

a Severe disease: HIV group had 74 patients, transplant group had 18 patients and 39 patients were in the HIV-negative/non-transplant group; Non-severe disease: HIV group had 12 patients, transplant group had 24 patients and 40 patients were in the HIV-negative/non-transplant group.

b Significantly associated with increased attributable-mortality.

c Cochran Mantel-Haenszel Chi-square test for a general association between the three groups.

In the HIV-negative/non-transplant group (n = 79), 34 (43%) had CNS disease, 38 (48%) had pulmonary cryptococcosis and 7 (9%) had another form of cryptococcosis (Table 1). There were 37 (47%) patients with no underlying malignancy or immunosuppressive therapy at the time of diagnosis. Ninety percent of all cancers in the cohort were among HIV-negative, non-transplant patients (Table 2). Of the 31 patients (39%) taking steroids prior to cryptococcosis diagnosis, 24 (77%) had dose information. The median daily dose was 20 mg prednisone or prednisone equivalent (range: 5–267 mg/day). The median duration of any type of immunosuppressive therapy (N = 21) was 7 months (IQR: 1–36 months). Eight patients (21%) with severe disease experienced a >50% decrease in GFR during induction treatment.

### Clinical Symptoms

There were 21 patients (10%) who were asymptomatic at the time of diagnosis (Table 2). Of the 19 with asymptomatic pulmonary disease, 10 were transplant recipients and nine were HIV-negative/non-transplant patients.

Among the patients reporting symptoms (n = 186), the duration of symptoms was unknown for 30 patients; 10 (12%) patients among those with HIV, seven (22%) of transplant recipients and 13 (18%) of the patients in the third group. Excluding these patients, the mean length of symptoms prior to presentation was not significantly different between severe and non-severe disease. The mean symptom duration was longer for the HIV-negative/non-transplant patients than either of the other two groups (Table 3). When compared to this group, the difference in means was −25 days (95% Confidence Limits [CL] −50, 1) for the HIV-positive patients and −20 days (95% CL −48, 8) for transplant recipients with severe disease. The differences in means were even greater when comparing the groups with non-severe disease (Table 3).

**Table 3:** Differences in mean duration of symptoms (days) reported prior to presentation among those reporting any symptom(s) of cryptococcosis (N = 156).

Underlying condition	Severe disease				Non-severe disease			
	n	Mean	Difference	95% CL^a^	n	Mean	Difference	95% CL^a^
HIV-negative, non-transplant	36	44	0 (ref.)	20, 68	21	66	0 (ref.)	29, 103
HIV positive	65	19	−25	−50, 1	10	26	−40	−80, 0
Transplant	14	24	−20	−48, 8	11	20	−46	−*84,* −*8*
**Total**	**114**	**28**		**19, 37**	**42**	**44**		**24, 64**

a Unadjusted 95% Confidence Limits (CL) of the difference in means. The 95% CL for the referent group and the total are surrounding their corresponding mean (days).

Patients with severe disease frequently experienced headaches, altered mental status, fevers, nausea and vomiting across all three groups at initial presentation (Table 2). The prevalence of nausea and vomiting was significant between groups with them being more common in HIV and transplant recipients. Furthermore, headache was significantly more prevalent among HIV-positive patients (73%) and similar between the other two groups. Symptoms among non-severe cryptococcosis patients were similar between transplant recipients and HIV-negative/non-transplant groups. However, the prevalence of corticosteroid exposure was significantly higher in transplant recipients (Table 2).

### Patient Diagnostics

Among patients with severe disease, patients had similar lumbar puncture (LP) results among all three groups (Table 4). At least one opening pressure (OP) measurement was available for 79 (63%) patients. Peak OP distributions were very similar between all three groups with a mean of 33 cm H_2_O (SD±16 cm H_2_O). The proportion of HIV-positive patients (48%) with elevated CSF host cells (≥20 cells/mm^3^) was significantly less than the other groups, which had frequencies of 78% and 61% (Table 4). The difference in frequencies across all three patient groups having an elevated CSF CRAG titer (≥1∶1024) and positive India ink neared significance. With regard to the frequency of these two CSF diagnostic measures, further comparison of non-HIV/non-transplant with the other two groups combined (differences in frequencies between HIV-positive and transplant patients were not significant) indeed reached the level of significance. CSF glucose and protein levels were similar in all groups (∼40% of all patients had hypoglycorrhachia). There was no significant difference between groups in regards to cryptococcemia (Table 4).

**Table 4:** Diagnostic findings of cryptococcosis disease (N = 207) stratified by disease severity at initial presentation and underlying condition.

		Severe disease (n = 131)^a^							Non-severe disease (n = 76)^a^						
Diagnostic	Result description	HIV		Transplant		HIV-/Trans-		*p<0.05	HIV		Transplant		HIV-/Trans-		*p<0.05
		n	(%)	n	(%)	n	(%)		n	(%)	n	(%)	n	(%)	
Positive culture															
	CNS, first LP^b^	61	(82)	18	(100)	22	(56)		0	-	0	-	0	-	
	Blood^c^	35	(47)	8	(44)	11	(28)		6	(50)	2	(8)	5	(13)	*
	Pulmonary	6	(8)	2	(11)	3	(8)		2	(17)	13	(54)	21	(53)	
Initial LP^b^															
	CSF CRAG titer ≥1:1024	35	(48)	6	(33)	8	(24)		-	-	-	-	-	-	
	CSF:serum glucose ratio <0.6	66	(89)	16	(89)	28	(85)		-	-	-	-	-	-	
	CSF glucose ≤40mg/dL	43	(58)	11	(61)	20	(61)		-	-	-	-	-	-	
	CSF protein ≥45mg/dL	66	(89)	16	(89)	32	(97)		-	-	-	-	-	-	
	Nucleated cells >20cells/mm^3,^ d	30	(48)	14	(78)	20	(71)	*							
	OP ≥20cm H_2_O^e^	34	(76)	3	(38)	17	(52)		-	-	-	-	-	-	
	Positive India Ink	41	(55)	9	(50)	11	(33)		-	-	-	-	-	-	
Other															
	Serum CRAG titer ≥1:1024	47	(64)	11	(61)	17	(44)		5	(42)	3	(13)	4	(10)	*
	Other histological evidence	9	(12)	3	(17)	6	(18)		0	-	15	(63)	27	(68)	*

a Severe disease: HIV group had 74 patients, transplant group had 18 patients and 39 patients were in the HIV-negative/non-transplant group; Non-severe disease: HIV group had 12 patients, transplant group had 24 patients and 40 patients were in the HIV-negative/non-transplant group.

b Lumbar puncture; 176 total patients had an LP. Six HIV-negative, non-transplant patients with severe disease did not receive an LP and the remaining 25 patients without an LP were non-severe cases (18 HIV-negative/non-transplant, six transplant recipients, and one HIV-positive patient). Percentages relevant to the LP procedure reflect missing observations.

c Significantly associated with attributable-mortality.

d Eleven (15%) HIV-infected patients had missing documentation of CSF host (nucleated) cells (overall N = 63); excluding the six with no initial LP, five HIV-negative, non-transplant were missing host cell counts (overall N = 28).

e Opening pressure; maximum LP OP was used for this variable, as initial LP OP was infrequently captured. Denominators for each group with severe disease were 74, 8, and 33, respectively.

Among non-severe disease patients, histological evidence of *Cryptococcus* was identified in over 60% of the two HIV-negative groups (Table 4). Positive pulmonary cultures were identified by at least one positive culture, biopsy or broncho-alveolar lavage in >50% of HIV-negative patients, whereas only 17% (n = 2) of HIV-positive patients had documentation of pulmonary disease. Of the 40 HIV-negative/non-transplant patients, 22 (55%) received an LP to rule out CNS disease, which was not significant compared to the 18 (75%) transplant recipients but was significant compared to the 11 (92%) HIV-positive patients who received an LP when non-severe disease was identified.

### Initial Treatment

**Figure 2:**
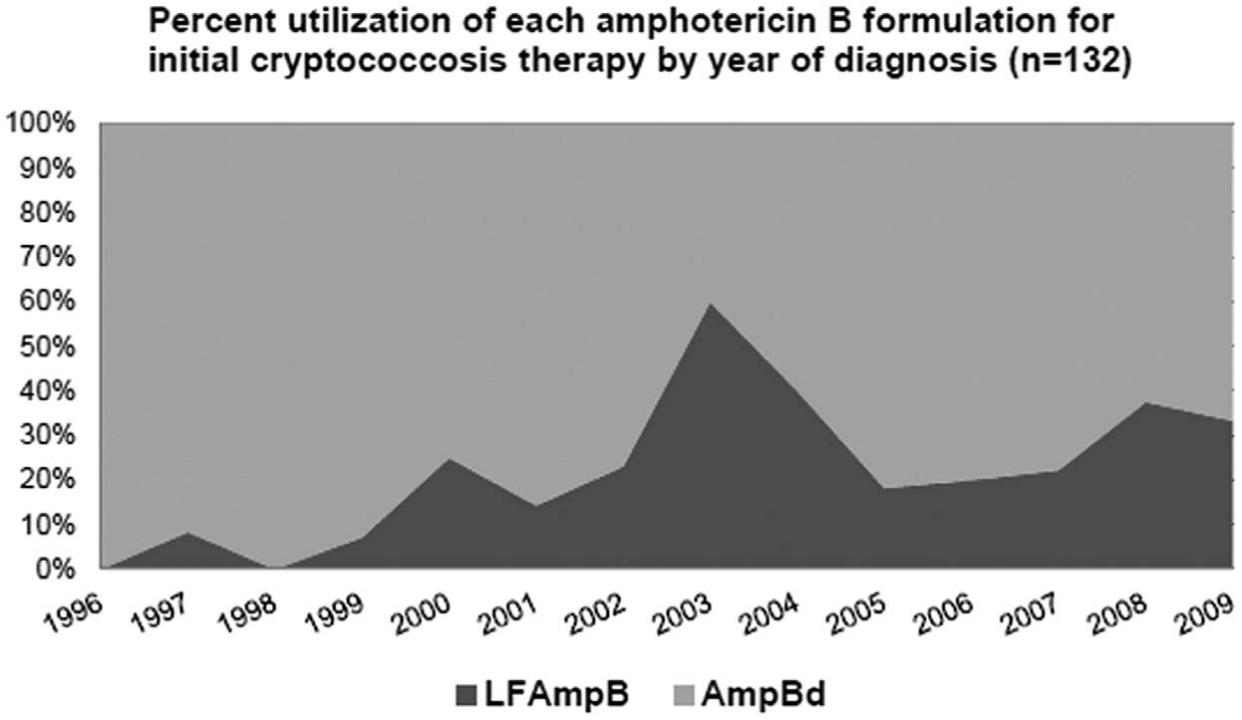
Use of amphotericin B. Amphotericin B formulation trends over time. Annual percentage of patients receiving lipid formulation amphotericin B (LF AmpB) or deoxycholate amphotericin B (AmpBd) for initial therapy (N = 132).

During the study period, 132 patients (64%) received amphotericin B (either formulation) for initial induction therapy. Utilization of amphotericin B deoxycholate (AmpBd) decreased over time, indicating that lipid formulation amphotericin B (LFAmpB) was used more frequently as initial therapy in recent years (Figure 2). Despite this observed trend, AmpBd was used as initial induction therapy for 80% of patients across the entire study period.

Induction antifungal regimens are summarized in Table 5. Eighty percent of patients with non-severe disease were given fluconazole for initial treatment; this was not significantly different across the three risk groups. In the severe disease group, the frequency of polyene use as initial therapy was high for all three patient groups (89% of HIV-positive, 100% of transplant, and 87% of HIV-negative/non-transplant), as was the use of flucytosine in combination with the polyene (78% of HIV-positive, 83% of transplant, and 72% of HIV-negative/non-transplant).

**Table 5:** Patient treatment and outcomes.

				Severe disease (n = 131)^a^						Non-severe disease (n = 76)^a^					
Outcomes	Description	Total		HIV		Transplant		HIV−/Trans-b		HIV		Transplant		HIV−/Trans-b	
		n	(%)	n	(%)	n	(%)	n	(%)	n	(%)	n	(%)	n	(%)
Induction															
	AmpBd alone	18	(9)	7	(9)	1	(6)	5	(13)	1	(8)	2	(8)	2	(5)
	AmpBd+5FC	88	(43)	55	(74)	5	(28)	22	(56)	1	(8)	0	(–)	5	(13)
	LFAmpB alone	7	(3)	1	(1)	2	(11)	1	(3)	0	(–)	1	(4)	2	(5)
	LFAmpB+5FC	19	(9)	3	(4)	10	(56)	6	(15)	0	(–)	0	(–)	0	(–)
	Fluconazole	71	(34)	8	(11)	0	(–)	2	(5)	10	(83)	21	(88)	30	(75)
	Voriconazole	1	(<1)	0	(–)	0	(–)	1	(3)	0	(–)	0	(–)	0	(–)
	None	3	(1)	0	(–)	0	(–)	2	(5)	0	(–)	0	(–)	1	(3)
Result^c^															
	Attributable death	31	(15)	12	(16)	3	(17)	12	(31)	1	(8)	0	(–)	3	(8)
	Overall mortality	52	(25)	15	(20)	5	(28)	16	(41)	2	(17)	1	(4)	13	(33)
	IRIS	7	(3)	3	(4)	2	(11)	1	(3)	1	(8)	0	(–)	0	(–)

Initial induction antifungal regimen, patient mortality through one year, and immune reconstitution inflammatory syndrome (IRIS) by primary disease diagnosis (severe or non-severe) and major underlying condition (HIV, transplant, or HIV-negative and non-transplant).

a Severe disease: HIV group had 74 patients, transplant group had 18 patients and 39 patients were in the HIV-negative/non-transplant group; Non-severe disease: HIV group had 12 patients, transplant group had 24 patients and 40 patients were in the HIV-negative/non-transplant group.

b HIV-negative, non-transplant.

c Cochran Mantel-Haenszel Chi-square test for a general association between the three groups was significant for overall mortality among non-severe disease only.

### Mortality and IRIS

Mortality attributable to cryptococcosis was 15% (n = 31) and there was a total of 52 deaths (25%) through one year of follow-up (Table 5). HIV-positive and HIV-negative/non-transplant patients accounted for 89% of severe disease cryptococcosis-attributable deaths and these two groups accounted for 86% of all-cause mortality. IRIS was identified in seven (3%) cases and most of these cases had severe cryptococcosis. Four out of the seven patients were HIV-positive but IRIS was observed in the other two groups.

The HIV-negative/non-transplant group experienced both greater mortality attributable to cryptococcosis and overall mortality, since they accounted for nearly half of all cryptococcosis-attributable deaths (15/31) and more than half of all-cause mortality (29/52). Within this group, patients who died were older at diagnosis (mean, 63 years) than those who did not (mean, 51 years). However, the average age at diagnosis was not significantly different between survivors and those who died within either the HIV-positive or transplant groups.

## Discussion

The 2010 IDSA Guidelines divided cryptococcal disease into three risk groups because of their different management issues in an attempt to better describe the issues around treatment and outcome [2]. The results from our study found notable trends and important clinical differences between and within these groups and uniquely describes the realities in the management of this disease at one institution.

In the early parts of the 14-year study period, the highest number of cases occurred in the HIV-infected population, which appeared to experience fewer of cryptococcal infections in recent years, coinciding with the widespread use of HAART. However, 42% of HIV-positive patients in this cohort had been exposed to HAART, emphasizing that despite therapies to control HIV infection, cryptococcosis will continue to be an opportunistic infection in HIV-infected persons. The HIV-negative/non-transplant patients appeared to offset the reduction of cryptococcosis seen in HIV-infected patients in more recent years. As we continue to aggressively treat serious underlying diseases with immunosuppressants and the denominator of persons-at-risk enlarges, this group will likely increase since there is no strategy for prophylaxis. There was a consistent number of cases of cryptococcosis in the transplant recipients over time despite the widespread use of the potentially anti-cryptococcal agents, the calcineurin inhibitors [34], [35], [36], [37], [38]. The steady annual prevalence of cryptococcosis in this group supports the continued routine use of immunosuppressants and thus a persistent need for careful diagnostic surveillance for detection of early cryptococcosis [39].

The differences within the groups were several. For HIV-infected individuals, there was a variety of antiretroviral strategies employed during anti-cryptococcal therapy that reflects the lack of precise guidelines on when to initiate HAART [40]. Similar to previous studies, most of these patients had profound CD4 lymphocytopenia and a majority of these patients (76%) had elevated intracranial pressure [16], [17], [41], [42]. The important underlying issues surrounding transplant recipients were immunosuppressive drugs and frequent renal dysfunction. All had some form of immunosuppression but only one-quarter had their immunosuppressive regimens stopped or changed and the prevalence of IRIS was low. Also, one-third of patients started treatment with evidence of renal dysfunction, emphasizing that lipid products of amphotericin B may be essential therapeutic choices in this group and that monitoring flucytosine levels and/or complete blood counts may be necessary to prevent treatment toxicity during worsening of renal dysfunction caused by polyene treatment [43]. Moreover, the average time from transplant to cryptococcal infection was within range of the 17–28 months reported in previous studies [37], [43], [44], [45], [46]. There were two important findings in the HIV-negative/non-transplant group. First, the duration of symptoms in this group with severe disease averaged 44 days prior to diagnosis and although not reaching significance from the other groups (it was significant among those with non-severe disease), this notable delay deserves greater attention and has been observed in a previous case series [23]. This delay may have contributed to the observed poorer outcome of the group. Another study also found a lack of significance between HIV-infected, immunocompromised, and immunocompetent groups, where the symptom duration averaged approximately 15 days [25]. It is possible that the other two groups have specialists aware of the risk of cryptococcosis, while in this group diagnosis is delayed because cryptococcosis is not considered. Second, 33% had liver insufficiency or hematological malignancies. These are important findings as this subgroup had the highest mortality and both factors have been shown to be predictors of mortality in HIV-negative cryptococcosis patients [24], [47]. Previous results emphasize that disseminated cryptococcosis among HIV-negative patients experienced the worst prognosis secondary to the stage of underlying disease and the immunosuppressive medication used [26], [34]. It has been clearly shown in the HIV-positive population with cryptococcal disease that stage of HIV is strongly associated with poor outcome [48]. The underlying disease and its stage are major factors in cryptococcosis outcome.

Since delay in diagnosis may be a prognostic factor, we investigated whether there were additional differences in symptoms and laboratory findings between the three groups. There were few differences between HIV-infected patients and transplant recipients who generally had similar symptoms and CSF laboratory parameters. However, headaches (known to be a prognostic factor) [29], were similar between both HIV-negative groups and significantly more prevalent in the HIV-positive group. While all three groups had a high prevalence of poor prognostic signs such as altered mental status (∼1/3), the presence of nausea and vomiting were less common in the HIV-negative/non-transplant group with severe disease. These findings differ from another study that reported significantly more mental status changes in non-immunosuppressed patients compared to HIV-positive patients [25]. Additionally, our study supports previous evidence that suggests non-immunosuppressed patients have less fungemia [24]. The HIV-negative/non-transplant group also appeared to present with a smaller burden of yeasts by India ink and CRAG test results than the other two groups. Although appreciation for burden of organisms and outcome could not be precisely understood in this retrospective review, prospective studies of cryptococcal meningitis may benefit from quantification of viable yeasts in the CSF (Colony-forming unit [CFU]/mL measurements) and understanding its rate of change during therapy in relationship to treatment strategy and outcome [12], [49], [50].

Importantly, compared to HIV-positive and transplant recipients, the attributable mortality in the HIV-negative/non-transplant population with severe disease was nearly two-times higher despite the fact that the majority of patients received induction therapy with a polyene and flucytosine. A recent multi-center study of 86 cryptococcal meningitis patients also found the highest mortality in the non-immunosuppressed group (46%) compared to immunosuppressed (19%) and HIV-positive (15%) cryptococcal meningitis patients [25], and previous studies have reported 30 - 44% overall mortality in the HIV-negative population [22], [23], [24], [26]. In one of these studies this rate was compared to a 22% mortality among HIV-positive patients [24]. However, a couple of these studies included some *C. gatii* cases so that species’ factors may have influenced outcome [22], [24]. Clearly more studies to inform the management of the HIV-negative/non-transplant group and to understand how host immunity and yeast strain may play a role in poorer prognosis are needed so as to reduce this elevated mortality.

One of the major complications of cryptococcosis management has been the identification and management of IRIS. We identified the occurrence of IRIS in all three groups but the prevalence (3%) was relatively low compared to other studies which primarily included AIDS patients (range, 8–19%) [51], [52], [53], [54]. This frequency could be influenced by our definition of IRIS, the patient mix, and/or clinical management. In general, our HAART management was delayed (>60 days after start of induction therapy) and only approximately a quarter of the transplant recipients had their immunosuppressants adjusted. This lack of immune manipulation during early induction therapy may influence our lower rate of IRIS. However, it is identified in a measureable amount of all patient groups and needs to be appreciated by clinicians.

Lipid formulations of amphotericin B outside of HIV-infected patients have limited critical appraisal of proper dosing and efficacy [55], [56]. However, as Figure 2 demonstrated, there is a general increase in the use of lipid formulations for induction therapy at our institution. We expect this was in relationship to the approximately third of patients who develop nephrotoxicity during management. Therefore, further investigation into lipid products of amphotericin B is still warranted to ensure their optimal use.

### Limitations

This review was limited to a single tertiary care center and teaching hospital. Our medical center averaged nearly 15 cases of cryptococcosis per year and this likely reflects both an endemic exposure to this yeast in the environment within the Southeastern USA and an enriched population of immunosuppressed individuals due to our hospital’s care patterns. The actual number of cases seen in a particular medical center certainly varies within the U.S. Furthermore, using hospital records favors cases with severe disease and could result in selection bias against asymptomatic disease. Determining the total population-at-risk was not estimable in this study and the underlying source population and referral patterns could shift over time. Retrospective chart review has the potential for incomplete or incorrect information capture due to loss of paper documentation or lack of entry in electronic medical record. We used both sources to ensure data was as complete as possible and discrepancies were minimized. Erroneous self-report of symptoms or symptom duration was a possibility, but this is a limitation of many observational clinical studies. Despite our careful abstraction process, missing or incomplete data could lead to bias in categorization of symptoms or derived outcome definitions, such as IRIS. Being a rare disease, limited numbers of cases prevented robust statistical analyses, although this study is one of the largest to date. Importantly, much of the clinical attention over the last two decades has centered around two groups (HIV-infected and transplant recipients). There has been less focus on treatment of HIV-negative/non-transplant patients and yet this group suffered the highest mortality.

### Conclusions

In summary, dividing patients with cryptococcosis into three risk groups showed both differences and similarities within the groups. In a single medical center the overall frequency of cryptococcosis has not changed though the composition of the three groups has changed in the last two decades. Despite three major classes of drugs to treat severe disease and a relatively uniform and informed treatment strategy framed by the IDSA Guidelines, attributable mortality was common. Prospective multi-center studies and comparison of strategies in advanced medical centers are still needed to determine the extent high mortality revolved around underlying disease, high burden of organisms and delayed diagnosis.
