# Correction: Comparison and Temporal Trends of Three Groups with Cryptococcosis: HIV-Infected, Solid Organ Transplant, and HIV-Negative/Non-Transplant

**DOI:** 10.1371/annotation/a94bc542-6682-4579-a315-57019cef7e0e

**Published:** 2012-10-30

**Authors:** Emily W. Bratton, Nada El Husseini, Cody A. Chastain, Michael S. Lee, Charles Poole, Til Stürmer, Jonathan J. Juliano, David J. Weber, John R. Perfect

There were errors in the author affiliations. The affiliations should appear as shown here:

Emily W. Bratton^1*^, Nada El Husseini**^2^**, Cody A. Chastain**^3^**, Michael S. Lee**^4^**, Charles Poole**^1^**, Til Stürmer**^1^**, Jonathan J. Juliano**^5^**, David J. Weber**^1,5^**, John R. Perfect**^2^**

**1** Department of Epidemiology, University of North Carolina at Chapel Hill, Chapel Hill, North Carolina, United States of America, **2** Department of Medicine, Duke University, Durham, North Carolina, United States of America, **3** Department of Medicine, Vanderbilt University Medical Center, Nashville, Tennessee, United States of America, **4** Department of Medicine, Brigham and Women’s Hospital, Boston, Massachusetts, United States of America, **5** Department of Medicine, University of North Carolina at Chapel Hill, Chapel Hill, North Carolina, United States of America 

